# Acupuncture for poststroke spasticity: A protocol of a systematic review and meta-analysis

**DOI:** 10.1097/MD.0000000000017124

**Published:** 2019-09-27

**Authors:** Li-hong Shi, Liu-xue Guo, Hui-ling Zhang, Yu-xi Li, Dong-ling Zhong, Qi-wei Xiao, Juan Li, Xiao-qian Ye, Rong-jiang Jin

**Affiliations:** a School of Health Preservation and Rehabilitation, Chengdu University of Traditional Chinese Medicine; b Affiliated Hospital of Chengdu University of Traditional Chinese Medicine; c School of Acupuncture-Moxibustion and Tuina, Chengdu University of Traditional Chinese Medicine, Chengdu, Sichuan; d School of Acupuncture, Fujian University of Traditional Chinese Medicine, Fujian, China.

**Keywords:** acupuncture, meta-analysis, protocol, spasticity, stroke, systematic review

## Abstract

**Introduction:**

Spasticity is the most common complication after stroke, which is the main obstacle in the recovery of motor function. Spasticity seriously affects the quality of life and brings a heavy burden to families and society. Acupuncture is an effective method for stroke. However, whether acupuncture is effective for poststroke spasticity is still unknown. The purpose of this systematic review (SR) is to evaluate the effectiveness and safety of acupuncture for poststroke spasticity.

**Methods and analysis:**

We will search the following databases from inception to July 2019: China Biology Medicine (CBM), China National Knowledge Infrastructure (CNKI), Wan Fang Data, the Chinese Science and Technology Periodical Database (VIP), PubMed, Embase, The Cochrane Library, and Web of Science. All relevant randomized controlled trials (RCTs) utilizing acupuncture for poststroke spasticity will be included. The primary outcome is the modified Ashworth scale. Secondary outcomes include composite spasticity scale, clinic spasticity index, electromyographic activity, Hoffmann reflex activity, or other spasticity-related outcomes. Study selection, data extraction, and quality assessment will be performed independently by 2 reviewers. Assessment of risk of bias and data synthesis will be conducted using Review Manager V5.3 software.

**Ethics and dissemination:**

The ethical approval is not required since SR is based on published studies. The results of this SR will be published in a peer-reviewed scientific journal according to the Preferred Reporting Item for Systematic Review and Meta-analysis (PRISMA) guidelines.

**PROSPERO registration number:**

CRD42019129779.

## Abstract 2

Supplemental Digital Content is available in the text

## 1 Introduction

Stroke is defined as an acute local injury of the central nervous system which caused by blood vessel lesions, such as cerebral infarction, cerebral hemorrhage, or subarachnoid hemorrhage.^[1]^ As one of the leading causes of disability and mortality worldwide, stroke is the major cause of death in China,^[2,3]^ while the most common poststroke complication is spasticity,^[4]^ with the prevalence ranging from 30% to 80% of stroke survivors.^[5]^ Spasticity, a motor disorder, characterized by a velocity-dependent increase in tonic stretch reflexes with exaggerated tendon spasms resulting in hyperarousal of the stretch reflex, which is a component of upper motor neuron syndrome.^[6]^ Spasticity is often associated with limb pain, joint contracture, and may lead to abnormal movement patterns,^[7]^ which can further hinder the recovery of motor function and participation in activities of daily living.^[8]^

Medications, surgical interventions and physical therapy are common treatments for poststroke spasticity, but the long-term treatment of spasticity with high cost has become a financial burden to patients and society.^[9–14]^ The long treatment period may lead to poor patient compliance, which greatly reduces the therapeutic effect.^[15]^ In addition, drugs and surgical treatments have some potential adverse reactions.^[16]^ Thus, we need a low cost, effective, and accessible method that has the potential to help reducing spasticity and promoting recovery. Acupuncture, a key component of traditional Chinese medicine, is an effective method and recommended for stroke according to the World Health Organization.^[17,18]^ A considerable number of clinical studies have demonstrated that acupuncture can relieve poststroke spasticity.^[19–28]^

The previous existing 3 systematic reviews (SRs) had a common limitation of poor methodological quality and none of these SRs reported study protocols in advance.^[29–31]^ Park et al^[29]^ did not use comprehensive literature search strategy. Lim et al^[30]^ did not evaluate the safety of acupuncture for poststroke spasticity. Cai et al^[31]^ mainly focused on the add-on effect of electroacupuncture. Overall, there is a lack of supportive evidence on the effectiveness and safety of acupuncture (including all types of acupuncture) for spasticity in patients with stroke. The aim of this study is to systematically review current available literature to assess the effectiveness and safety of the acupuncture for poststroke spasticity.

## 2 Methods and analysis

### 2.1 Study registration

The protocol of this SR has been registered in PROSPERO (registration number: CRD42019129779). This SR will be reported following the Preferred Reporting Item for Systematic Review and Meta-analysis (PRISMA) statement guidelines.

### 2.2 Inclusion criteria

#### 2.2.1 Type of studies

Only randomized controlled trials (RCTs) of acupuncture for poststroke spasticity will be included without any restriction on language or publication status.

#### 2.2.2 Type of participants

Poststroke patients (over 18 years old) with spasticity will be included. There will be no restriction on gender, race, or nation.

#### 2.2.3 Type of interventions

The RCTs that used acupuncture to treat poststroke spasticity will be included. There will be no limit on the types of acupuncture.

#### 2.2.4 Type of comparators

The comparative interventions could be usual care, conventional rehabilitations, sham acupuncture, no treatment, or other active treatments.

#### 2.2.5 Types of outcome measurements

The primary outcome will be the modified Ashworth scale. Secondary outcomes will include composite spasticity scale, clinic spasticity index, electromyographic activity, Hoffmann reflex activity, or other spasticity-related outcomes. In addition, adverse events will also be assessed as safety measurement.

### 2.3 Exclusion criteria

The exclusion criteria include:

1. Cluster-RCTs, cross-over trials, observational studies, case reports, reviews
2. Spasticity caused by other diseases
3. Acupuncture combined with other treatments (except usual care and conventional rehabilitation)
4. Duplicated publications or the data cannot be extracted
5. Full text cannot be obtained through various approaches

### 2.4 Search strategy

We will search the following databases from inception to July 2019: China Biology Medicine (CBM), China National Knowledge infrastructure (CNKI), Wan Fang Data, the Chinese Science and Technology Periodical Database (VIP), PubMed, Embase, The Cochrane Library, and Web of Science, using the combination of key words of acupuncture, stroke, spasticity, and RCT. References of identified relevant RCTs will be searched manually for relevant literatures. We will not apply any language or date restrictions. The search strategy of PubMed is shown in Appendix 1.

### 2.5 Studies selection

**Figure 1:**
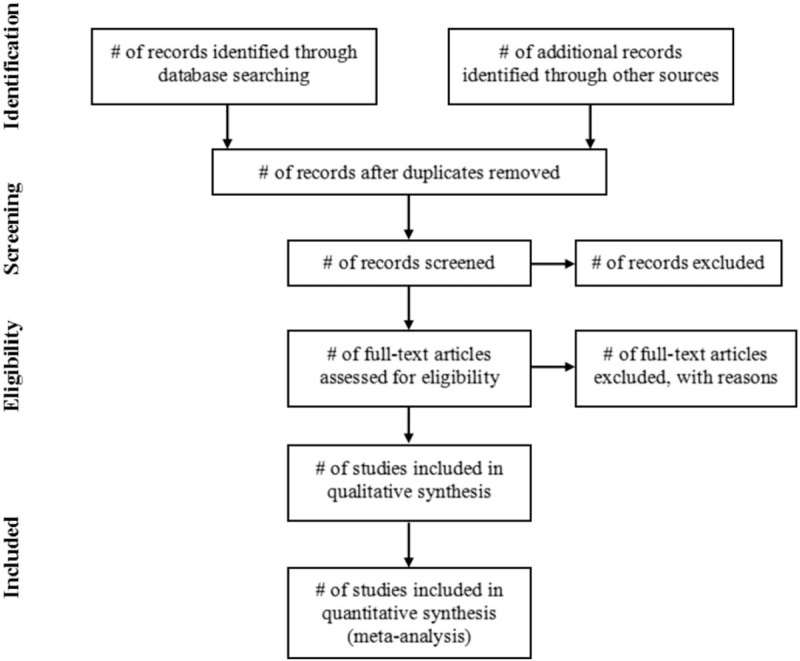
PRISMA flowchart.

All the retrieved studies will be managed with Endnote X9, and the duplicated studies will be filtered. Two reviewers (LHS and YXL) will independently screen the studies by titles and abstracts according to the predefined inclusion criteria. Then 2 reviewers will download the full texts of all possibly relevant studies and further examine full-text reports independently. Two reviewers will cross check the included studies. Disagreements will be resolved by discussion or consensus with a 3rd reviewer (RJJ). The procedures of study selection will be performed in accordance with the PRISMA flowchart (see Fig. 1).

### 2.6 Data extraction

The following information will be extracted by 2 reviewers (DLZ and QWX) independently with a predesigned data extraction form: general characteristics (1st author, year of publication, language, and country of study), participant characteristics (sample size, mean age, sex, course of disease), intervention characteristics (type, frequency, duration), and outcomes (primary and secondary outcomes, adverse events). If necessary, the authors will be contacted for more information to supplement the missing data. As for divergences, a 3rd reviewer (JL) will be involved in.

### 2.7 Assessment of risk of bias

Two reviewers (LXG and HLZ) will independently assess the risk of bias for included studies using the Cochrane Handbook for Systematic Reviews of Interventions tool. The following risk of bias domains will be assessed: random sequence generation, allocation concealment, blind subjects and therapists, blind assessors, incomplete outcome data, selective outcome reporting, and other bias. Each domain will be considered as low (meet all criteria)/unclear (trials with insufficient information to judge)/high risk (meet none of the criteria) of bias. In case of opposition, a 3rd reviewer (XQY) will be involved in.

### 2.8 Data analysis

RevMan 5.3 software provided by the Cochrane collaboration will be used to perform a meta-analysis. We will use the relative risk to analyze dichotomous outcomes, while the mean difference or standardized mean difference will be used to analyze continuous outcomes. The uncertainty will be expressed with 95% confidence intervals. We will measure heterogeneity using the *I*^2^ statistic. Fixed-effects model will be used if there is no statistical heterogeneity among studies (*I*^2^ < 50%, *P* > .1). Otherwise, random-effects model will be used. When meta-analysis is not possible, we will perform narrative analysis.

#### 2.8.1 Dealing with missing data

If the data of included study is lacking, insufficient, or unclear, the original authors will be contacted via email for more information. If there were no reply from the original authors, we will only analyze the available data and discuss the potential impact of those missing data in the text.

#### 2.8.2 Subgroup analysis

To investigate heterogeneity, we will conduct subgroup analyses based on age, sex, type of stroke (hemorrhagic and ischemic stroke), type of acupuncture (acupuncture, electroacupuncture, warming acupuncture, etc), region of spasticity (upper limbs, lower limbs), treatment frequency, and treatment duration.

#### 2.8.3 Sensitivity analysis

To check the robustness of pooled outcome results, we will carry out sensitivity analysis to evaluate the impact of studies with high risk of bias.

#### 2.8.4 Publication bias

When a meta-analysis includes 10 or more RCTs, funnel plot will be performed to evaluate the publication bias. We will try to interpret funnel plot asymmetry if funnel plots are asymmetric.

### 2.9 Grading of Recommendations Assessment, Development and Evaluation

We will use Grading of Recommendations Assessment, Development, and Evaluation (GRADE) system to evaluate the quality of evidence of outcomes. Each outcome will be assessed from the 5 aspects: limitations, inconsistency, indirectness, imprecision, and publication bias.^[32]^ The quality of evidence will be rated as “high,” “moderate,” “low,” or “very low.” The results of GRADE will be shown in evidence profile and summary of finding table using GRADE prosoftware.

### 2.10 Ethics and dissemination

No ethical approval is required because this SR will be performed based on published studies. The results of this SR will be published in a peer-reviewed scientific journal according to the PRISMA guidelines.

## 3 Discussion

Acupuncture is widely used for spasticity after stroke. Numerous studies have concluded that acupuncture can relieve spasticity.^[33–36]^ At present, the researches on the antispasmodic mechanism of acupuncture mainly focus on the effect of acupuncture on spasm-related neurotransmitters and receptors, relieving spasticity by increasing the expression of inhibitory transmitters or decreasing the expression of excitatory neurotransmitters.^[37,38]^ Some studies have found that acupuncture promotes the recovery of damaged neurons in brain tissue and senior central functions by reducing the expression level of inflammatory factors and regulating cell signal transmission, so as to establish a normal spinal cord reflex mechanism and relieve muscle spasm.^[39–43]^ In addition, acupuncture has been proved to protect central neurons by improving blood supply in ischemic areas of the brain and promoting the proliferation of central nerve cells, so as to achieve the functional restructuring of the central nervous system,^[44,45]^ which is crucial for strengthening the central control of lower motor neurons to regulate muscle tension and relieve muscle spasm. However, there is still lack of valid evidence to support that acupuncture is effective for poststroke spasticity. Therefore, we will conduct an SR and meta-analysis to assess the effectiveness and safety of acupuncture for poststroke spasticity. It is hoped that the results of this SR may help to establish a better approach for the treatment of poststroke spasticity and provide reliable evidence for its wide application.

### Strengths and limitations



This SR will provide a comprehensive assessment regarding the effectiveness and safety of acupuncture for spasticity in patients poststroke, including all types of acupuncture. The protocol of this SR has been registered in PROSPERO. The SR will be conducted and reported in strict accordance with the standards in the AMSTAR2.0 and PRISMA. However, there may be a limitation with search, since only English and Chinese databases will be searched.

## Author contributions

**Data curation:** Yu-xi Li, Dong-ling Zhong.

**Methodology:** Juan Li, Xiao-qian Ye, Rong-jiang Jin.

**Resources:** Qi-wei Xiao.

**Supervision:** Liu-xue Guo, Hui-ling Zhang.

**Writing – original draft:** Li-hong Shi.

## Supplementary Material

Supplemental Digital Content

## References

[1] Sacco RL Kasner SE Broderick JP An updated definition of stroke for the 21st century: a statement for healthcare professionals from the American Heart Association/American Stroke Association. Stroke 2013;44:2064–89. 23652265 10.1161/STR.0b013e318296aeca PMC11078537

[2] Rajsic S Gothe H Borba HH Economic burden of stroke: a systematic review on post-stroke care. Eur J Health Econ 2019;20:107–34. 29909569 10.1007/s10198-018-0984-0

[3] Wu S Wu B Liu M Stroke in China: advances and challenges in epidemiology, prevention, and management. Lancet Neurol 2019;18:394–405. 30878104 10.1016/S1474-4422(18)30500-3

[4] Lee SH Wong YO Chu MK Acupuncture in managing of spasticity of post stroke survivor: a systematic review. Stroke 2012;43:A3506.

[5] Kuo CL Hu GC Post-stroke spasticity: a review of epidemiology, pathophysiology, and treatments. Int J Gerontol 2018;12:280–4.

[6] Lance JW The control of muscle tone, reflexes, and movement: Robert Wartenberg Lecture. Neurology 1980;30:1303–13. 7192811 10.1212/wnl.30.12.1303

[7] Baricich A Picelli A Molteni F Post-stroke spasticity as a condition: a new perspective on patient evaluation. Funct Neurol 2016;31:179–80. 27678212 10.11138/FNeur/2016.31.3.179 PMC5115233

[8] Thibaut A Chatelle C Ziegler E Spasticity after stroke: physiology, assessment and treatment. Brain Inj 2013;27:1093–105. 23885710 10.3109/02699052.2013.804202

[9] Sun LC Chen R Fu C Efficacy and safety of botulinum toxin type A for limb spasticity after stroke: a meta-analysis of randomized controlled trials. Biomed Res Int 2019;8329306. 31080830 10.1155/2019/8329306 PMC6475544

[10] Lindsay C Kouzouna A Simcox C Pharmacological interventions other than botulinum toxin for spasticity after stroke. Cochrane Database Syst Rev 2016;10:CD010362. 27711973 10.1002/14651858.CD010362.pub2 PMC6457886

[11] Xiang J Wang W Jiang W Effects of extracorporeal shock wave therapy on spasticity in post-stroke patients: a systematic review and meta-analysis of randomized controlled trials. J Rehabil Med 2018;50:852–9. 30264850 10.2340/16501977-2385

[12] Kwong PW Ng GY Chung RC Transcutaneous electrical nerve stimulation improves walking capacity and reduces spasticity in stroke survivors: a systematic review and meta-analysis. Clin Rehabil 2018;32:1203–19. 29232981 10.1177/0269215517745349

[13] Tamburella F Moreno JC Iosa M Boosting the traditional physiotherapist approach for stroke spasticity using a sensorized ankle foot orthosis: a pilot study. Top Stroke Rehabil 2017;24:447–56. 28460597 10.1080/10749357.2017.1318340

[14] Wang J Yu P Zeng M Reduction in spasticity in stroke patient with paraffin therapy. Neurol Res 2017;39:36–44. 27876449 10.1080/01616412.2016.1248169

[15] Lu TT Yang WM Clinical research progress of limb spasm after cerebral apoplexy [in Chinese]. Chin J Integr Med Cardiocerebrovasc Dis 2017;15:2263–8.

[16] Yang CY Xu W Li G Development of drug therapy for hemiplegia and spasm after stroke [in Chinese]. Chin J Med Guide 2011;13:1516–7.

[17] Bonafede M Dick A Noyes K The effect of acupuncture utilization on healthcare utilization. Med Care 2008;46:41–8. 18162854 10.1097/MLR.0b013e3181589b7d

[18] Wang J Pei J Khiati D Acupuncture treatment on the motor area of the scalp for motor dysfunction in patients with ischemic stroke: study protocol for a randomized controlled trial. Trials 2017;18:287. 28633675 10.1186/s13063-017-2000-x PMC5479040

[19] Li H Long D Li B A clinical study to assess the influence of acupuncture at “Wang's Jiaji” acupoints on limb spasticity in patients in convalescent stage of ischemic stroke: study protocol for a randomized controlled trial. Trials 2019;20:419. 31291976 10.1186/s13063-019-3464-7 PMC6621988

[20] Wang HQ Hou M Bao CL Effects of acupuncture treatment on lower limb spasticity in patients following hemorrhagic atroke: a pilot study. Eur Neurol 2019;81:5–12. 31013499 10.1159/000499133

[21] Sánchez-Mila Z Salom-Moreno J Fernández-de-Las-Peñas C Effects of dry needling on post-stroke spasticity, motor function and stability limits: a randomised clinical trial. Acupunct Med 2018;36:358–66. 29986902 10.1136/acupmed-2017-011568

[22] Qi L Han Z Zhou Y Dynamic scalp acupuncture combined with PNF therapy for upper limb motor impairment in ischemic stroke spastic hemiplegia [in Chinese]. Zhongguo Zhen Jiu 2018;38:234–8. 29701038 10.13703/j.0255-2930.2018.03.002

[23] Yang J Xiao H The effect of floating-needle therapy combined with rehabilitation training for the hand function recovery of post-stroke patients [in Chinese]. Zhongguo Zhen Jiu 2015;35:758–62. 26571885

[24] Han SK Zhang BC Zuo YF Observations on the efficacy of muscle-region alignment needling plus skin acupuncture in treating post-stroke upper limb spasticity [in Chinese]. Shanghai J Acupunct Moxibust 2010;29:284–6.

[25] Fan LB Liu SZ Wang ZT Application of electro-acupuncture plus movement therapy in recovering neurologic function of patients with spastic hemiplegia [in Chinese]. Shanghai J Acupunct Moxibust 2015;34:1178–80.

[26] Wang N Li Z Life quality improvement of spastic hemiplegia of stroke treated with fire-needle: a randomized controlled trial [in Chinese]. Zhongguo Zhen Jiu 2015;35:1105–9. 26939318

[27] Liu M Li ZH Ma H Clinical evaluation of electric acupuncture at antagonistic muscle acupoints combined with rehabilitation training for the treatment of apoplexy spastic paralysis [in Chinese]. J Clin Acupunct Moxibust 2016;32:8–10.

[28] Huang RL Xia Q Zhu ZJ Clinical effect of acupuncture combined with rehabilitation training in treatment of hemiplegia spasticity after stroke [in Chinese]. J Anhui Univ Chin Med 2016;35:59–62.

[29] Park SW Yi SH Lee JA Acupuncture for the treatment of spasticity after stroke: a meta-analysis of randomized controlled trials. J Altern Complement Med 2014;20:672–82. 25192034 10.1089/acm.2014.0097 PMC4155415

[30] Lim SM Yoo J Lee E Acupuncture for spasticity after stroke: a systematic review and meta-analysis of randomized controlled trials. Evid Based Complement Alternat Med 2015;2015:870398. 25628750 10.1155/2015/870398 PMC4299539

[31] Cai Y Zhang CS Liu S Electroacupuncture for poststroke spasticity: a systematic review and meta-analysis. Arch Phys Med Rehabil 2017;98:2578–89. 28455191 10.1016/j.apmr.2017.03.023

[32] GRADE Working Group. Grading quality of evidence and strength of recommendations. BMJ 2004;328:1490. 15205295 10.1136/bmj.328.7454.1490 PMC428525

[33] Mukherjee M McPeak LK Redford JB The effect of electro-acupuncture on spasticity of the wrist joint in chronic stroke survivors. Arch Phys Med Rehabil 2007;88:159–66. 17270512 10.1016/j.apmr.2006.10.034

[34] Yue ZH Li L Ye Y Effect of acupuncture on content of serum amino acid in spasticity paralysis after stroke [in Chinese]. J Trad Chin Med Univ Hunan 2010;30:212–5.

[35] Xu L Wang M Li F Acupuncture combined with rehabilitation training for the limb spasm after stroke [in Chinese]. Zhongguo Zhen Jiu 2017;37:696–700. 29231540 10.13703/j.0255-2930.2017.07.004

[36] Dall’Agnol MS Cechetti F Kinesio taping associated with acupuncture in the treatment of the paretic upper limb after stroke. J Acupunct Meridian Stud 2018;11:67–73. 29436374 10.1016/j.jams.2017.12.003

[37] Liu C Li R Song X Effect of catgut implantation at acupoints on GABA(B) and mGluR1 expressions in brain stem of rats with spasticity after stroke. J Tradit Chin Med 2014;34:566–71. 25417407 10.1016/s0254-6272(15)30064-9

[38] He T Yan CQ Zeng XH Effects of acupuncture on amino acid content in the striatum and spinal cord in MCAO rats [in Chinese]. Chin J Trad Chin Med Pharm 2015;30:3105–7.

[39] Wang S Cong Y Study on the effect of acupuncture with different meridian points on serum IP_3_ and DAG levels in spasm rats after cerebral apoplexy [in Chinese]. Chin J Trad Med Sci Technol 2012;19:52–4.

[40] Wang S Zhang J Study on the effect of acupuncture with different meridian points on serum cAMP and cGMP levels in spasm rats after cerebral apoplexy [in Chinese]. Chin J Trad Med Sci Technol 2012;19:54–5.

[41] Wang S Hu YH Effects of acupuncture with different meridians on the expression of PKC in cerebral tissue of spasm rats after stroke [in Chinese]. Chin J Trad Med Sci Technol 2012;19:519–21.

[42] Sang P Wang S Zhao JH Effects of acupuncture with different meridians on the expression of PDGF-B and PDGFR-β in cerebral tissue of spasm rats after stroke [in Chinese]. Chin J Trad Med Sci Technol 2012;19:524–5.

[43] Qi YC Xiao XJ Duan RS Effect of acupuncture on inflammatory cytokines expression of spastic cerebral palsy rats. Asian Pac J Trop Med 2014;7:492–5. 25066401 10.1016/S1995-7645(14)60081-X

[44] Li J He J Du Y Electroacupuncture improves cerebral blood flow and attenuates moderate ischemic injury via Angiotensin II its receptors-mediated mechanism in rats. BMC Complement Altern Med 2014;14:441. 25387826 10.1186/1472-6882-14-441 PMC4237754

[45] Hong J Wu G Zou Y Electroacupuncture promotes neurological functional recovery via the retinoic acid signaling pathway in rats following cerebral ischemia-reperfusion injury. Int J Mol Med 2013;31:225–31. 23129018 10.3892/ijmm.2012.1166

