# Supplementary material for: Acupuncture for poststroke spasticity: A protocol of a systematic review and meta-analysis
Source: Medicine (Baltimore). 2019 Sep 27;98(39):e17124. doi: 10.1097/MD.0000000000017124 (PMC6775425; doi:10.1097/MD.0000000000017124)
Supplement: Supplemental Digital Content [file medi-98-e17124-s001.doc]

**Appendix 1**

**Search strategy for Pubmed：**

#1 Cerebrovascular Disorders[Mesh]

#2 cerebrovascular disorders[Title/Abstract] OR brain ischemia[Title/Abstract] OR intracranial hemorrhages[Title/Abstract] OR cerebral hemorrhage[Title/Abstract] OR cerebrovascular trauma[Title/Abstract] OR SAH[Title/Abstract] OR brain infarction[Title/Abstract] OR stroke[Title/Abstract]

#3 #1 OR #2

#4 Muscle Hypertonia[Mesh]

#5 muscle hypertonia[Title/Abstract] OR muscle spasticity[Title/Abstract] OR muscle rigidity[Title/Abstract] OR muscle tonus[Title/Abstract] OR muscle cramp[Title/Abstract] OR dystonia[Title/Abstract] OR spasm*[Title/Abstract] OR spastic*[Title/Abstract]

#6 #4 OR #5

#7 Acupuncture Therapy[Mesh]

#8 acupuncture therapy[Title/Abstract] OR acupuncture*[Title/Abstract] OR electroacupuncture[Title/Abstract] OR electro-acupuncture[Title/Abstract]

#9 #7 OR #8

#10 randomized controlled trial[Publication Type]

#11 randomized controlled trial[Title/Abstract] OR random*[Title/Abstract] OR placebo[Tittle/Abstract]

#12 #10 OR #11

#13 #3 AND #6 AND #9 AND #12
